# Supplementary material for: High-throughput DNA extraction and cost-effective miniaturized metagenome and amplicon library preparation of soil samples for DNA sequencing
Source: PLoS One. 2024 Apr 4;19(4):e0301446. doi: 10.1371/journal.pone.0301446 (PMC10994328; doi:10.1371/journal.pone.0301446)
Supplement: S1 Fig — (PDF) [file pone.0301446.s001.pdf]

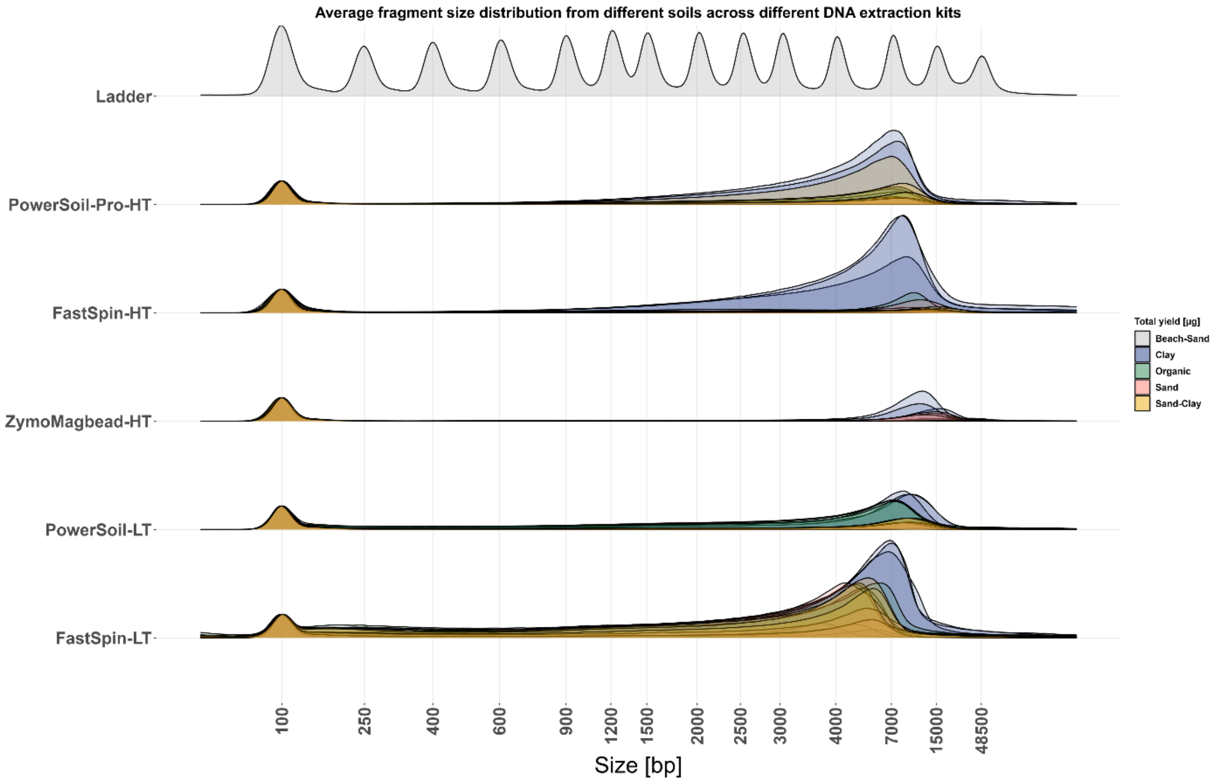

S1 Fig. Genomic DNA fragment distribution across different DNA extraction kits. Each replicate is colored by soil type.
