# Supplementary material for: High-throughput DNA extraction and cost-effective miniaturized metagenome and amplicon library preparation of soil samples for DNA sequencing
Source: PLoS One. 2024 Apr 4;19(4):e0301446. doi: 10.1371/journal.pone.0301446 (PMC10994328; doi:10.1371/journal.pone.0301446)
Supplement: S2 Fig — (PDF) [file pone.0301446.s002.pdf]

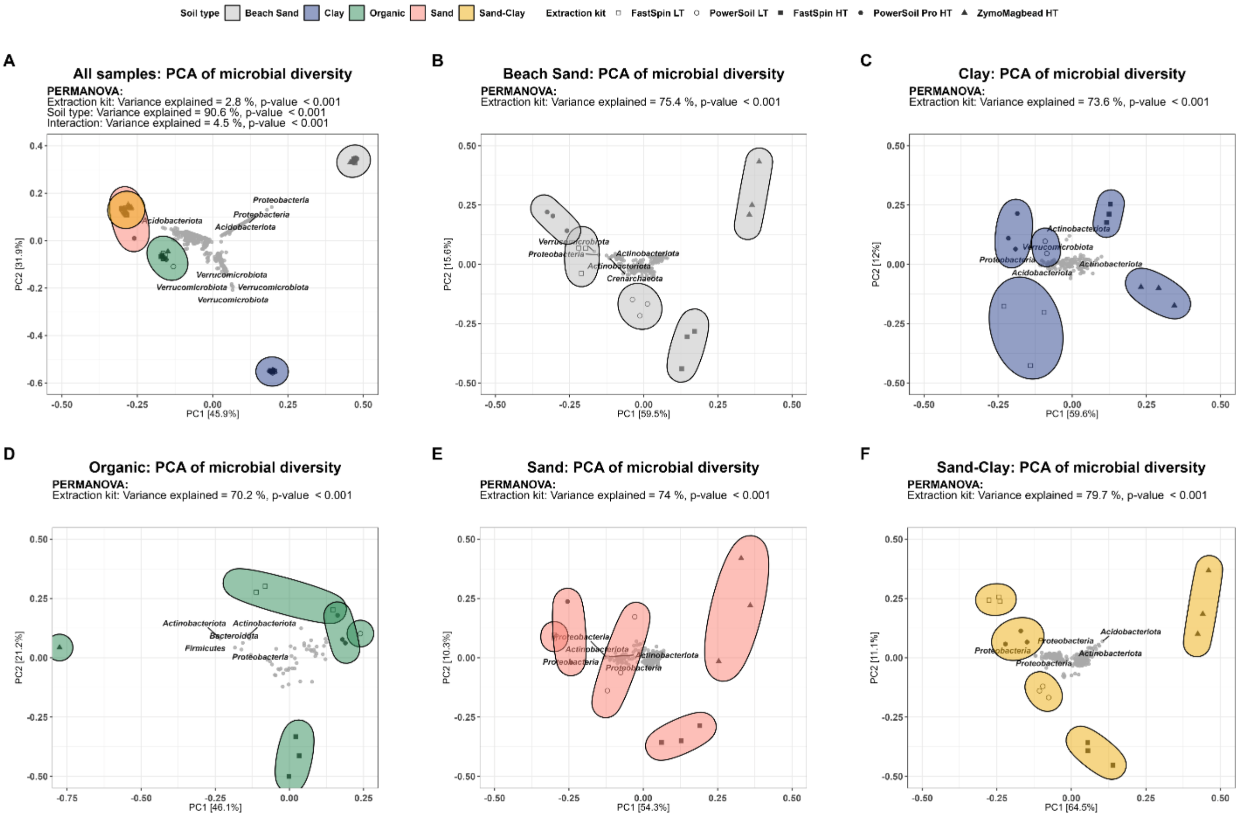

S2 Fig. PCA of DNA extraction kits stratified by soil type. (A) Beach Sand, (B) Clay, (C) Organic, (D) Sand, (E) Sand-Clay. OTUs not exceeding 0.1% relative abundance in at least one sample were removed before Hellinger-transformation.
