# Supplementary material for: High-throughput DNA extraction and cost-effective miniaturized metagenome and amplicon library preparation of soil samples for DNA sequencing
Source: PLoS One. 2024 Apr 4;19(4):e0301446. doi: 10.1371/journal.pone.0301446 (PMC10994328; doi:10.1371/journal.pone.0301446)
Supplement: S3 Fig — (PDF) [file pone.0301446.s003.pdf]

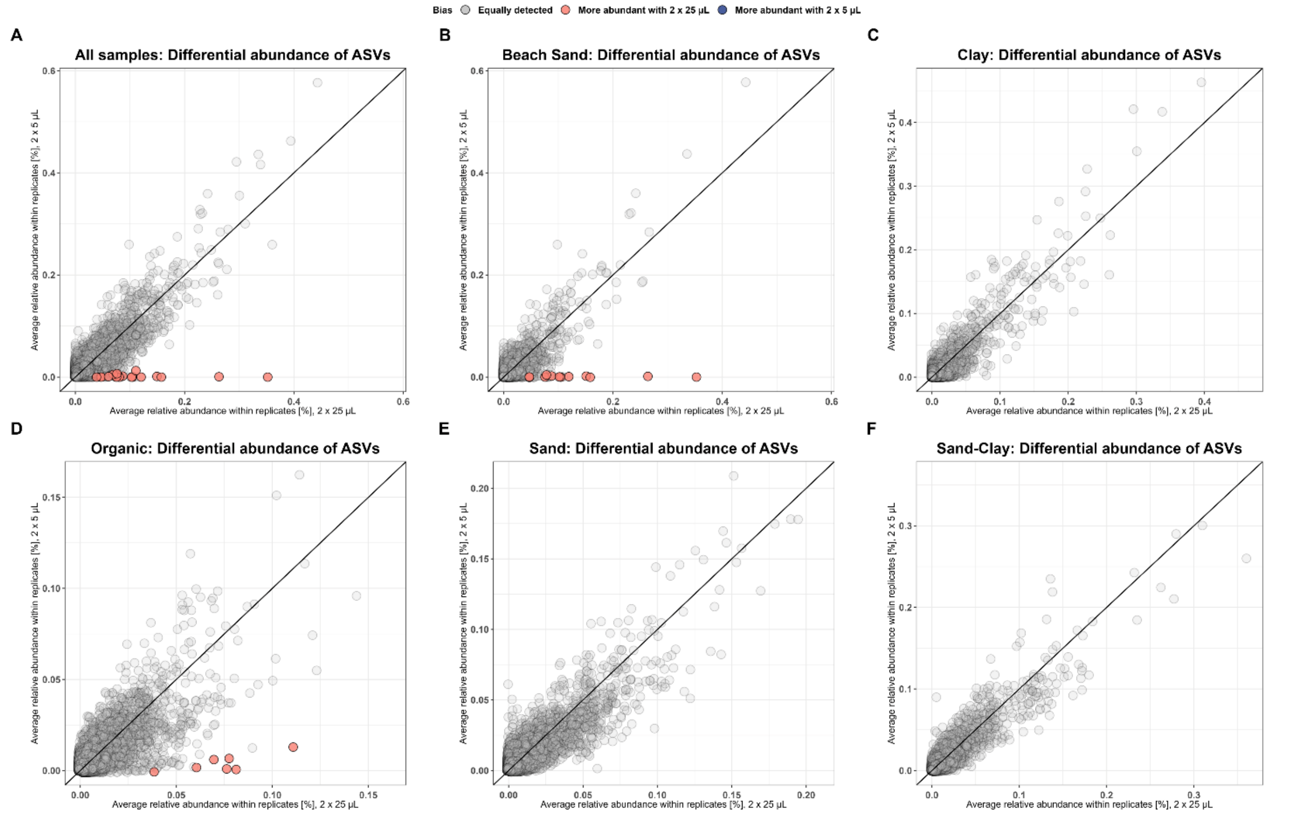

S3 Fig. Differential abundance plot for each soil type. (A) Differential abundance plot for each soil type: (B) Beach Sand, (C) Clay, (D) Organic, (E) Sand, (F) Sand-Clay. ASVs differentially abundant in one protocol are marked with color. Bias was calculated with DESeq2 and is defined as a significant difference in log2-fold-change (adjusted p-value<0.05).
