# Supplementary material for: High-throughput DNA extraction and cost-effective miniaturized metagenome and amplicon library preparation of soil samples for DNA sequencing
Source: PLoS One. 2024 Apr 4;19(4):e0301446. doi: 10.1371/journal.pone.0301446 (PMC10994328; doi:10.1371/journal.pone.0301446)
Supplement: S4 Fig — (PDF) [file pone.0301446.s004.pdf]

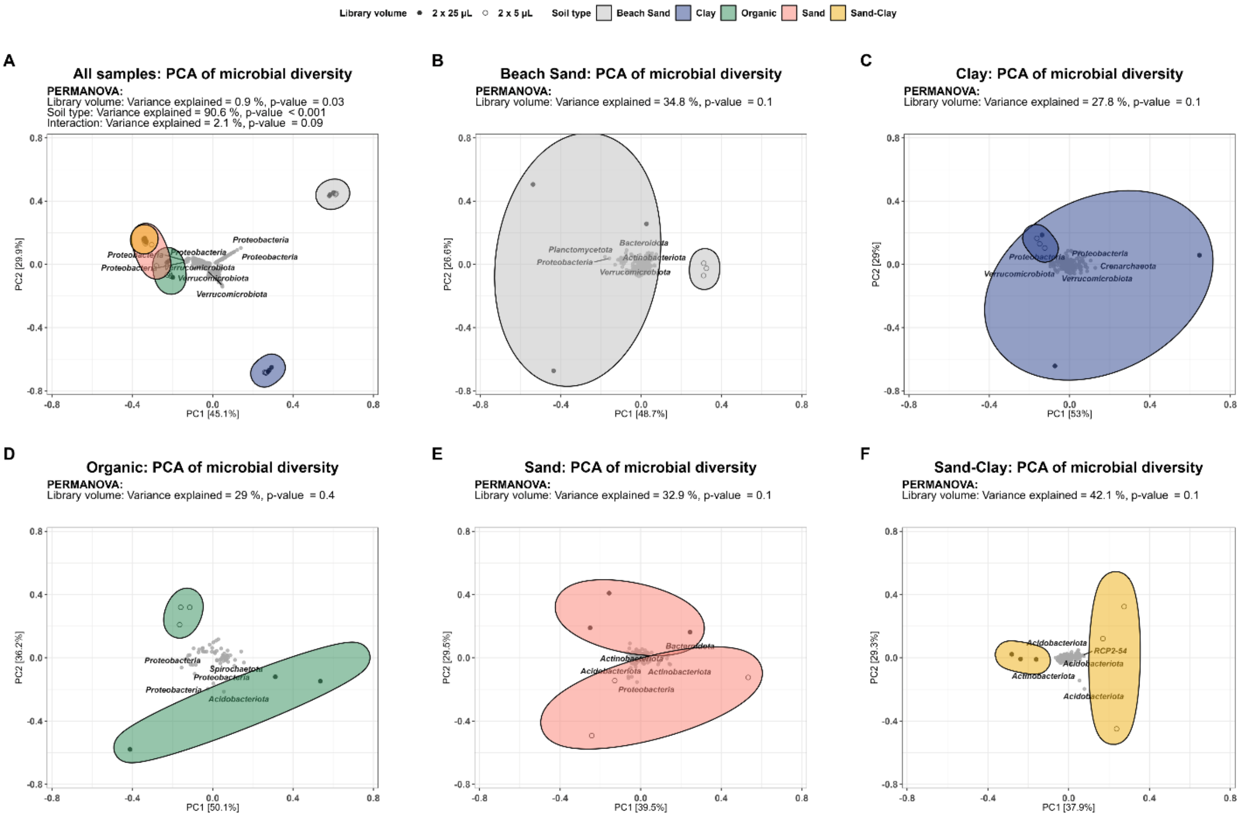

S4 Fig. PCA of the miniaturized and standard protocol for amplicons. (A) PCA of miniaturized and standard amplicon protocol. PCA of miniaturized and standard amplicon protocol stratified by soil type: (B) Beach Sand, (C) Clay, (D) Organic, (E) Sand, (F) Sand-Clay. ASVs not exceeding 0.1% relative abundance in at least one sample were removed before Hellinger-transformation.
