# Supplementary material for: High-throughput DNA extraction and cost-effective miniaturized metagenome and amplicon library preparation of soil samples for DNA sequencing
Source: PLoS One. 2024 Apr 4;19(4):e0301446. doi: 10.1371/journal.pone.0301446 (PMC10994328; doi:10.1371/journal.pone.0301446)
Supplement: S1 Table — (PDF) [file pone.0301446.s010.pdf]

Sample	Site name	Subsamples	Natura 2000	Habitat	Longitude,Latitude	pH	Humic load
Organic	Gravlev Kær	5	7230	Alkaline fens	56.8330,9.8202	4.44	Extreme
Sand	Urskoven	5	9110/9120	Beech forests	56.8154,9.8359	4.04	High
Sand-Clay	Rold	5	9110/9120	Beech plantation	56.8138,9.8510	3.68	High
Clay	Hestehaven	5	9130	Beech forests	56.2853,10.4733	5.80	Intermediate
Beach Sand	Kalø	5	1220	Perennial vegetation of stony banks	56.2732,10.4699	8.90	Low

S1 Table. Sample characteristics. Soil sample characteristics as well as location of sample site.
