# Supplementary material for: High-throughput DNA extraction and cost-effective miniaturized metagenome and amplicon library preparation of soil samples for DNA sequencing
Source: PLoS One. 2024 Apr 4;19(4):e0301446. doi: 10.1371/journal.pone.0301446 (PMC10994328; doi:10.1371/journal.pone.0301446)
Supplement: S2 Table — (PDF) [file pone.0301446.s011.pdf]

		Laboratory metrics (n=3)				Sequencing Metrics (n=Libraries)					
Soil type	Extraction Kit	DNA yield [µg]	260/280	260/230	Integrity [kbp]	Libraries	Library conc. [ng/µL]	Number of reads	Observed ASVs	Shannon Diversity	Bray-Curtis Dissimilarity
Beach Sand	FastSpin LT	NA*	1.90 (0.09)	0.02 (0.00)	4.78 (1.00)	3	14.29 (11.18)	37941 (17752)	5931 (15)	8.26 (0.02)	0.08 (0.01)
	PowerSoil LT	0.09 (0.01)	1.48 (0.18)	0.70 (0.19)	10.69 (0.71)	3	25.96 (2.62)	44685 (11229)	5739 (211)	8.22 (0.11)	0.12 (0.03)
	FastSpin HT	0.02 (0.00)	3.17 (0.43)	0.18 (0.01)	13.76 (1.14)	3	27.57 (2.08)	49568 (5044)	5918 (32)	8.29 (0.02)	0.08 (0.00)
	PowerSoil Pro HT	0.16 (0.03)	1.79 (0.17)	0.95 (0.04)	7.22 (0.95)	3	10.15 (0.97)	39084 (10378)	5498 (64)	8.18 (0.01)	0.11 (0.01)
	ZymoMagbead HT	0.11 (0.02)	1.44 (0.31)	0.03 (0.02)	21.09 (5.60)	3	19.45 (1.53)	44464 (14380)	5604 (175)	8.22 (0.04)	0.10 (0.02)
Clay	FastSpin LT	NA*	1.75 (0.04)	0.26 (0.01)	6.92 (0.11)	2	12.76 (11.46)	46242 (5127)	6496 (70)	8.31 (0.03)	0.07 (NA)
	PowerSoil LT	2.99 (0.23)	1.88 (0.01)	1.95 (0.01)	8.68 (0.71)	3	20.30 (0.98)	46931 (12289)	6903 (88)	8.46 (0.03)	0.09 (0.01)
	FastSpin HT	2.91 (0.61)	2.08 (0.02)	0.67 (0.03)	8.76 (0.40)	3	24.89 (2.23)	36345 (17564)	6386 (132)	8.27 (0.05)	0.09 (0.01)
	PowerSoil Pro HT	4.20 (2.02)	1.92 (0.01)	2.15 (0.02)	6.87 (0.32)	3	18.46 (2.46)	41219 (13895)	6665 (67)	8.36 (0.02)	0.07 (0.00)
	ZymoMagbead HT	1.08 (0.27)	1.65 (0.17)	0.07 (0.06)	13.00 (2.26)	3	22.68 (1.05)	40117 (7998)	6662 (166)	8.39 (0.04)	0.09 (0.00)
Organic	FastSpin LT	NA*	1.56 (0.02)	0.38 (0.07)	5.45 (0.67)	1	5.94 (10.19)	35883 (NA)	8008 (NA)	8.76 (NA)	NA (NA)
	PowerSoil LT	2.82 (0.22)	1.87 (0.01)	1.89 (0.03)	6.78 (0.06)	3	20.75 (1.38)	54391 (11029)	8281 (49)	8.81 (0.01)	0.13 (0.01)
	FastSpin HT	1.08 (0.33)	2.88 (0.48)	0.21 (0.08)	17.16 (5.66)	3	16.88 (11.17)	40582 (11119)	7600 (356)	8.70 (0.06)	0.18 (0.01)
	PowerSoil Pro HT	1.75 (0.10)	1.93 (0.02)	1.80 (0.36)	7.04 (0.23)	3	15.51 (2.60)	37852 (5176)	8117 (155)	8.78 (0.04)	0.16 (0.01)
	ZymoMagbead HT	0.07 (0.07)	1.55 (0.24)	0.06 (0.01)	11.18 (2.56)	1	7.20 (12.40)	51355 (NA)	7455 (NA)	8.64 (NA)	NA (NA)
Sand	FastSpin LT	NA*	1.62 (0.04)	0.14 (0.04)	5.24 (0.69)	3	17.94 (1.27)	35966 (12534)	6885 (15)	8.52 (0.01)	0.11 (0.01)
	PowerSoil LT	0.77 (0.02)	1.78 (0.06)	1.43 (0.18)	8.31 (0.60)	3	21.90 (2.18)	34231 (11585)	6802 (112)	8.49 (0.03)	0.10 (0.01)
	FastSpin HT	0.71 (0.21)	2.65 (0.26)	0.26 (0.04)	11.09 (0.82)	2	23.48 (0.72)	44452 (1237)	6447 (16)	8.40 (0.02)	0.09 (NA)
	PowerSoil Pro HT	1.38 (0.26)	1.91 (0.01)	1.59 (0.38)	7.59 (0.63)	2	19.84 (6.02)	48816 (2551)	6798 (107)	8.50 (0.02)	0.13 (NA)
	ZymoMagbead HT	0.41 (0.15)	1.68 (0.10)	0.03 (0.01)	13.65 (1.16)	3	21.04 (3.39)	42188 (8694)	6396 (88)	8.37 (0.03)	0.10 (0.01)
Sand-Clay	FastSpin LT	NA*	1.53 (0.03)	0.26 (0.04)	5.45 (0.41)	3	18.60 (2.10)	45471 (5312)	6710 (74)	8.46 (0.01)	0.08 (0.00)
	PowerSoil LT	1.09 (0.05)	1.78 (0.03)	1.48 (0.11)	8.68 (0.20)	3	19.42 (2.04)	43166 (10862)	6584 (34)	8.43 (0.01)	0.08 (0.00)
	FastSpin HT	0.80 (0.09)	2.51 (0.10)	0.23 (0.02)	15.39 (2.51)	3	26.02 (2.07)	46872 (8983)	6185 (55)	8.30 (0.01)	0.08 (0.01)
	PowerSoil Pro HT	1.25 (0.16)	1.90 (0.04)	1.67 (0.13)	7.76 (0.93)	3	15.66 (0.74)	32527 (17196)	6506 (173)	8.39 (0.04)	0.10 (0.02)
	ZymoMagbead HT	0.03 (0.01)	1.38 (0.12)	0.03 (0.01)	21.28 (4.34)	3	11.14 (7.10)	28814 (2419)	6096 (139)	8.31 (0.05)	0.13 (0.00)

S2 Table. General statistics from DNA extraction, library preparation, and community profiles based on 16S rRNA amplicon data. All samples were rarefied to 11,468 reads (the lowest read count in any sample with more than 10,000 total reads). ASVs not exceeding 0.1 % relative abundance in at least one sample were removed prior to Hellinger-transformation and calculation of Bray-Curtis dissimilarity. Numbers represent mean and numbers in parentheses represent standard deviation. *DNA concentration could not be determined due to interference from humic substances.
