# Supplementary material for: High-throughput DNA extraction and cost-effective miniaturized metagenome and amplicon library preparation of soil samples for DNA sequencing
Source: PLoS One. 2024 Apr 4;19(4):e0301446. doi: 10.1371/journal.pone.0301446 (PMC10994328; doi:10.1371/journal.pone.0301446)
Supplement: S3 Table — (PDF) [file pone.0301446.s012.pdf]

Soil type	Protocol	Libraries	Library conc. [ng/µL]	Read Count	Observed ASVs	Shannon Diversity	Bray-Curtis Dissimilarity	BC Dissimilarity Between Protocols
Beach Sand	2 x 25 µL	3	3.92 (1.20)	4314 (834)	2394 (15)	7.60 (0.02)	0.29 (0.00)	0.34 (0.02)
	2 x 5 µL	3	10.15 (0.97)	39084 (10378)	2368 (27)	7.57 (0.02)	0.30 (0.00)	
Clay	2 x 25 µL	3	8.84 (7.48)	7414 (5359)	2538 (65)	7.65 (0.04)	0.30 (0.01)	0.30 (0.02)
	2 x 5 µL	3	18.46 (2.46)	41212 (13895)	2499 (15)	7.61 (0.01)	0.28 (0.01)	
Organic	2 x 25 µL	3	14.69 (2.23)	17742 (2182)	2798 (96)	7.83 (0.05)	0.48 (0.07)	0.48 (0.03)
	2 x 5 µL	3	15.51 (2.60)	37851 (5175)	2919 (35)	7.90 (0.02)	0.46 (0.03)	
Sand	2 x 25 µL	3	12.65 (1.11)	18601 (2987)	2566 (70)	7.71 (0.04)	0.34 (0.01)	0.36 (0.01)
	2 x 5 µL	3	19.84 (6.02)	36365 (21638)	2672 (38)	7.76 (0.02)	0.36 (0.03)	
Sand-Clay	2 x 25 µL	3	10.52 (1.56)	18993 (2423)	2524 (33)	7.66 (0.02)	0.30 (0.01)	0.33 (0.01)
	2 x 5 µL	3	15.66 (0.74)	32526 (17195)	2528 (76)	7.67 (0.05)	0.33 (0.02)	

S3 Table. General library characteristics of the miniaturized and standard amplicon protocol. Microbial community characteristics of the standard and miniaturized amplicon library protocol for five different soil. All samples were rarefied to 3,511 reads (the lowest read count in any sample with more than 3,000 total reads). ASVs not exceeding 0.1 % relative abundance in at least one sample were removed prior to Hellinger-transformation and calculation of Bray-Curtis dissimilarity. Numbers represent mean (n="Libraries") and numbers in parentheses represent standard deviation. The number of comparisons for "Bray-Curtis dissimilarity between protocols" was 9.
